# Induced Membrane Reconstruction of a 6‐cm Ulnar Defect With Concurrent Revision Bone Grafting of Radial Nonunion Following Gustilo–Anderson Type IIIB Open Forearm Fracture: A Case Report

**DOI:** 10.1155/cro/3342262

**Published:** 2026-10-01

**Authors:** John N. Manfredi, Abdullah B. Chandasir, Mitchell Lomis, Brent Prenger, Humzah Ali, Kyra Urquhart-Foster, Caleb J. Bischoff, Abrar Adil Ansari

**Affiliations:** ^1^ Medical College of Georgia Augusta Georgia USA gru.edu; ^2^ University of Georgia Athens Georgia USA uga.edu; ^3^ Department of Orthopedics Research University of Missouri School of Medicine Columbia Missouri USA missouri.edu; ^4^ OhioHealth Doctors Hospital Columbus Ohio USA; ^5^ Missouri Orthopedic Institute Columbia Missouri USA; ^6^ Department of Orthopedic Surgery Piedmont Athens Regional Medical Athens Georgia USA

**Keywords:** fracture-related infection, induced membrane technique, Masquelet technique, oral antibiotic suppression, radial nonunion, radioulnar variance, septic nonunion, *Serratia marcescens*, ulnar bone defect

## Abstract

The induced membrane technique (IMT) is a two‐stage reconstructive procedure for critical‐sized bone defects. We report a 31‐year‐old male who sustained a Gustilo–Anderson grade IIIB open fracture of the radius and ulna following a high‐energy dirt bike injury, complicated by *Serratia marcescens* fracture‐related infection and septic nonunion. Intraoperative debridement at Stage 1 revealed a 6‐cm defect of the ulna, which was managed with an antibiotic‐impregnated polymethylmethacrylate (PMMA) spacer. A concomitant radial nonunion, identified on postdebridement CT, was addressed at Stage 2 with concurrent revision open reduction and internal fixation (ORIF) and autogenous femoral bone grafting. Culture‐directed oral antibiotic therapy was administered following Stage 1. Mature bridging callus was noted at 6 months and confirmed at both bones at approximately 22 months postinjury, with infection eradication confirmed at Stage 2. Prior to subsequent elbow procedures, the patient demonstrated a Mayo Elbow Performance Score of 65 (fair), with an elbow arc of motion of 60°–140° and forearm rotation of 70° pronation and 75° supination. This case illustrates the utility of IMT for ulnar reconstruction in the setting of fracture‐related infection, and highlights a forearm‐specific biomechanical consideration: Fixing the ulna at a set length via the IMT spacer construct while shortening the radius for nonunion compression may alter radioulnar variance, a single‐case observation that we hypothesize could have implications for DRUJ and elbow mechanics and warrants further study.

## 1. Introduction

The treatment of large bone defects remains a significant challenge in orthopedic trauma surgery. Critical‐sized bone defects are generally defined as lesions exceeding the innate capacity for spontaneous regeneration without surgical intervention. Such defects require specialized reconstructive modalities, including vascularized bone grafts, distraction osteogenesis, or the induced membrane technique (IMT) [1, 2]. Whether arising from trauma, infection, or tumor resection, these defects necessitate restoration of both mechanical stability and a biologically favorable local environment for healing.

When bone defects are complicated by infection, eradication of the infectious nidus is a prerequisite to reconstruction. The current standard for fracture‐related infection with associated bone loss involves a staged approach: Radical debridement with antibiotic‐impregnated cement spacer placement, followed by definitive reconstruction once infection has been controlled and inflammatory markers have normalized [2, 3].

The IMT—also widely referred to in the literature as the Masquelet technique—is a two‐stage procedure in which a PMMA spacer is placed within the bone defect to occupy the dead space and elicit formation of a biologically active membrane [3]. First reported by Masquelet et al [4] in 2000, the technique was originally described in the context of complex reconstructive cases that incorporated soft tissue flap coverage as part of the operative sequence. Over time, the term “induced membrane technique” has emerged as the more mechanistically descriptive designation for the spacer‐membrane‐graft biology, and the two terms are now used interchangeably across the orthopedic literature. In cases where soft tissue coverage has been addressed independently—as with primary flap coverage at the index procedure—the IMT designation is applicable to the staged bone reconstruction that follows.

The biological basis of the induced membrane has been characterized through extensive basic science investigation. Implantation of the PMMA spacer triggers a foreign body reaction, initiating an inflammatory cascade with early infiltration of neutrophils and mesenchymal stem cells at the spacer surface. Over 4–6 weeks, this resolves into a mature, highly vascularized collagen‐based membrane approximately 1.6 mm thick in humans. The resulting structure elaborates bone morphogenetic protein‐2 (BMP‐2), vascular endothelial growth factor (VEGF), and transforming growth factor‐beta 1 (TGF‐*β*1), and serves a dual mechanical and biological role: Physically excluding fibrous soft tissue ingrowth while creating an osteogenic and angiogenic microenvironment that promotes incorporation of the subsequently placed bone graft [3, 5].

The preponderance of published IMT experience involves defects of the femur and tibia [3]. However, emerging reports demonstrate efficacy in the upper extremity. Walker et al. reported successful union in all nine patients with forearm defects at a median of 4.67 months [6], and Bourgeois et al. achieved union in five of six forearm cases at a mean of 4 months [7]. Cases involving IMT of the ulna with concurrent management of radial pathology at the same operative sitting remain rare in the literature.

We present a case of a 31‐year‐old male with a 6‐cm critical‐sized ulnar defect secondary to *Serratia marcescens* fracture‐related infection following a Gustilo–Anderson grade IIIB open forearm fracture, with a concomitant radial nonunion managed concurrently at Stage 2 via revision ORIF and bone grafting. We describe the antibiotic management strategy, the reconstructive approach, functional outcomes, and a forearm‐specific biomechanical consideration regarding radioulnar length discrepancy arising from the interplay between IMT spacer fixation of the ulna and radial shortening for nonunion compression.

## 2. Case Presentation

Written informed consent for publication of clinical details and imaging was obtained from the patient prior to preparation of this report. A structured patient timeline of the patient’s clinical course is presented in Figure 1.

**Figure 1:**
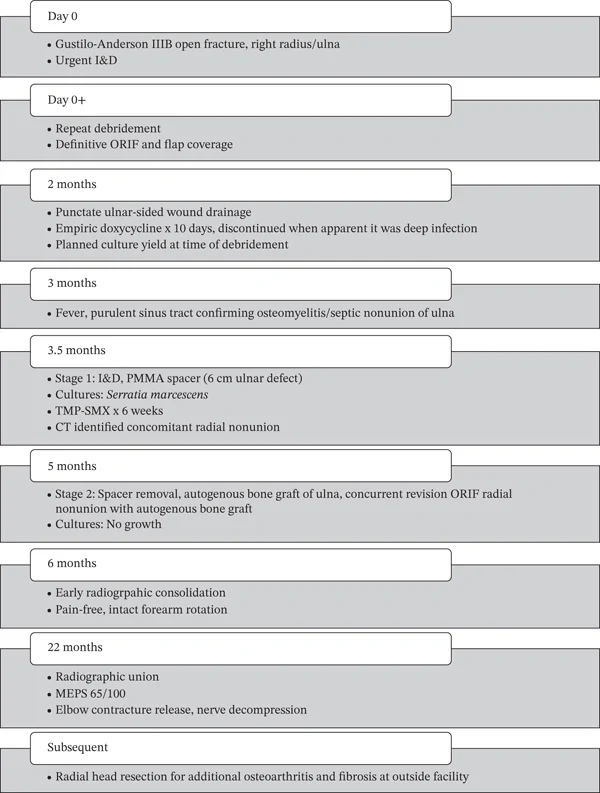
Clinical timeline illustrating the management of a Gustilo–Anderson type IIIB diaphyseal fracture of the radius and ulna complicated by *Serratia marcescens* osteomyelitis, treated with the Masquelet‐induced membrane technique.  ^∗^I&D, irrigation and debridement; ORIF, open reduction and internal fixation; PMMA, polymethylmethacrylate; TMP‐SMX, trimethoprim‐sulfamethoxazole; MEPS, Mayo Elbow Performance Score.

A 31‐year‐old male with no significant medical history presented to the emergency department via emergency medical services following a high‐energy dirt bike accident in a freshwater stream. He sustained a Gustilo–Anderson grade IIIB open diaphyseal fracture of the right radius and ulna with gross soil contamination (Figure 2). On the day of injury, urgent irrigation and debridement were performed by the on‐call orthopedic surgeon. Given the extent of contamination, repeat medullary debridement of both the radius and ulna was subsequently required. Definitive fixation with open reduction and internal fixation (ORIF) using plate osteosynthesis and simultaneous rotational fasciocutaneous flap coverage was performed (Figure 3).

**Figure 2:**
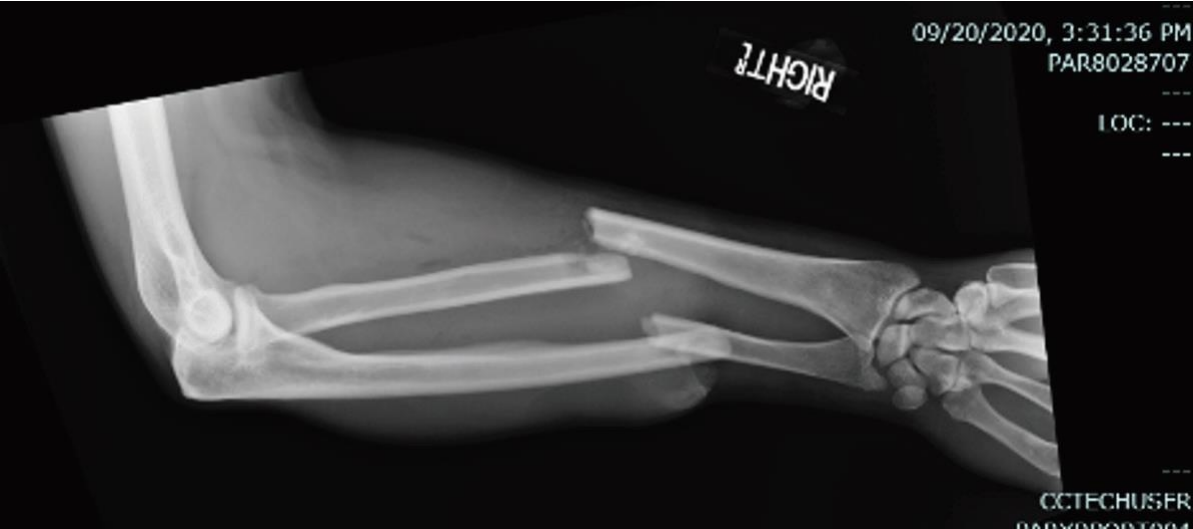
AP radiograph of the right forearm obtained on the day of injury, demonstrating a Gustilo–Anderson type IIIB diaphyseal fracture of the radius and ulna.

**Figure 3:**
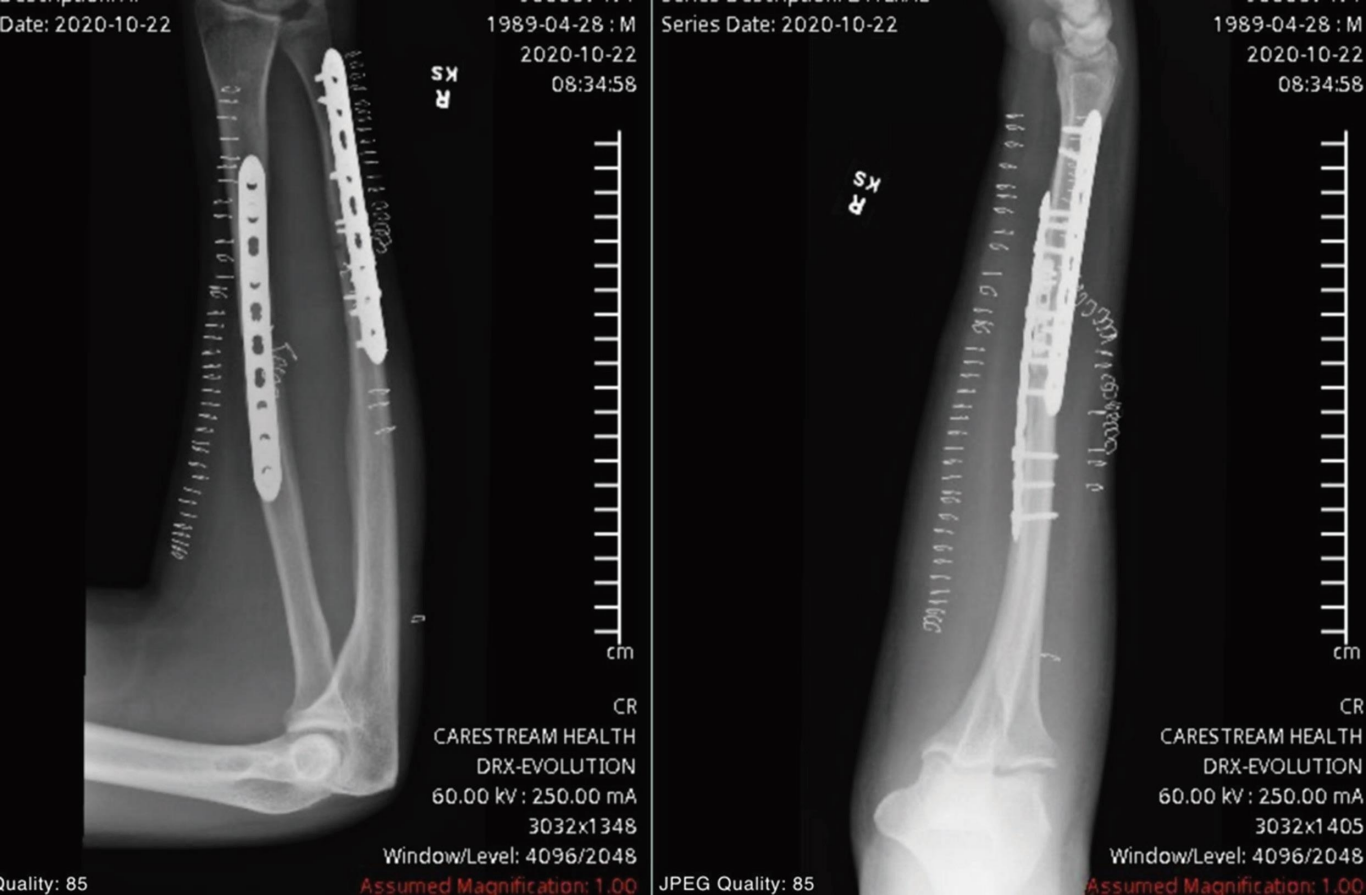
Postoperative AP and lateral radiographs taken approximately 1 month postinjury with plate osteosynthesis of the radius and ulna, and rotational fasciocutaneous flap coverage.

At approximately 2 months postinjury, the patient developed punctate ulnar‐sided wound drainage. Forearm range of motion remained appropriate, and the patient was pain‐free. Given the history of high‐energy contaminated open fracture and a wound of unknown depth, a 10‐day course of oral doxycycline 100 mg twice daily was initiated empirically. When clinical assessment established that this represented a deep infection rather than a superficial wound process, doxycycline was discontinued in order to optimize culture yield at the time of planned surgical debridement.

At approximately 3 months postinjury, the patient reported new‐onset fever and purulent discharge from a 1‐mm ulnar sinus tract, confirming the diagnosis of osteomyelitis and septic nonunion of the ulna. The patient underwent irrigation and debridement and Stage 1 of the IMT at approximately three and a half months postinjury. Intraoperatively, a 6‐cm critical‐sized ulnar bone defect was identified following radical debridement of all infected and necrotic tissue. An antibiotic‐impregnated PMMA spacer was prepared using Simplex P with tobramycin supplemented with vancomycin (1 g) and additional tobramycin (1.2 g) was placed within the ulnar defect to occupy the dead space and initiate membrane induction (Figure 4). A postdebridement CT scan identified a concomitant radial nonunion.

**Figure 4:**
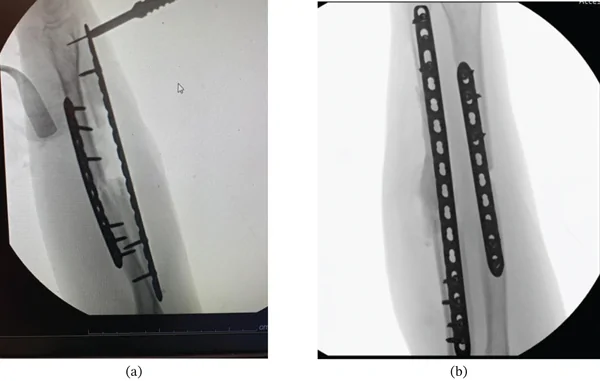
(a,b) Intraoperative fluoroscopic images obtained during Stage 1, demonstrating ulnar plate fixation following resection and PMMA spacer placement.

Intraoperative tissue cultures obtained at Stage 1 subsequently grew *S*. *marcescens* in two colony types. Susceptibility testing demonstrated sensitivity to trimethoprim‐sulfamethoxazole (TMP‐SMX), ciprofloxacin, gentamicin, levofloxacin, and tobramycin, with intrinsic resistance to ampicillin‐based agents and tetracycline—consistent with the known resistance profile of this organism. On the basis of these results, oral TMP‐SMX (Bactrim DS) 800/160 mg twice daily was initiated as culture‐directed therapy and continued for approximately 6 weeks post‐Stage 1, at which point inflammatory markers had normalized and wound healing was well established.

At approximately 5 months postinjury, the patient underwent Stage 2 of the IMT for the ulnar defect. The induced membrane was carefully incised and preserved, the PMMA spacer removed, and the ulnar defect packed with autogenous bone graft harvested from the ipsilateral proximal femur using medullary reamings harvested with intramedullary reamer‐irrigator‐aspirator. Concurrent revision ORIF of the radial nonunion with autogenous femoral bone graft was performed at the same operative sitting. This concurrent radial procedure represented standard revision bone grafting and fixation—not an application of the IMT, as no spacer had been placed for the radius. Intraoperative cultures obtained at Stage 2 showed no growth, confirming eradication of infection (Figures 5 and 6).

**Figure 5:**
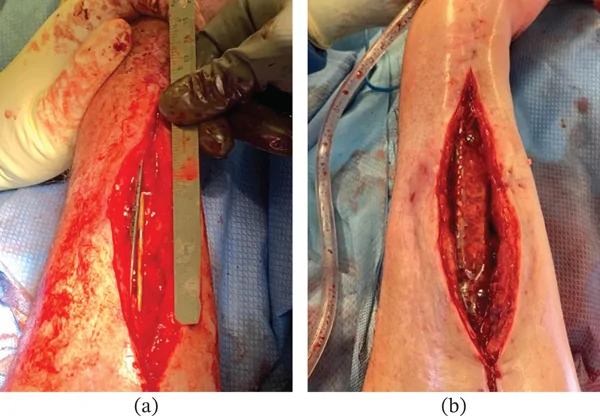
(a) Intraoperative photograph obtained during Stage 2 of the induced membrane technique, demonstrating removal of the spacer with the 6‐cm ulnar bone defect. An intramedullary elastic nail was placed to augment stability and maintain canal patency. (b) Intraoperative photograph obtained during Stage 2 of the induced membrane technique. Autogenous bone grafting and minifragment screws are visible securing the moldable polymer mesh poly‐L‐lactide‐co‐glycolide graft containment system.

**Figure 6:**
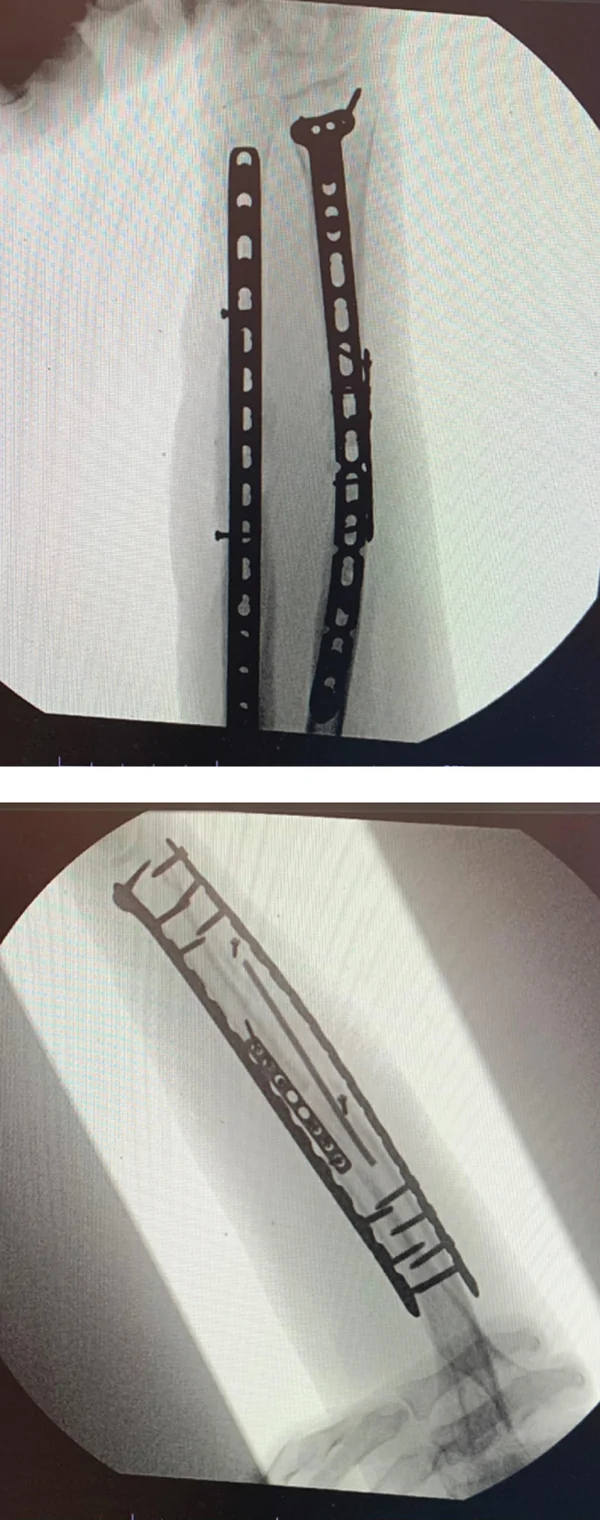
Intraoperative fluoroscopic views obtained during Stage 2 of the induced membrane technique.

At his first postoperative visit following Stage 2, the patient reported significant improvement with no forearm pain and only minimal donor site soreness requiring no opioid analgesia. Radiographs at approximately 6 months postinjury demonstrated early consolidation at the ulnar defect site without hardware loosening. Positive ulnar variance was noted on postoperative imaging compared with preoperative and intraoperative films, attributed to alteration of interosseous membrane tension as well as potential radial shortening performed during nonunion repair (Figure 7). The patient demonstrated no appreciable functional deficit in forearm rotation at this visit. Infection eradication was determined and confirmed by negative intraoperative cultures at Stage 2, normalization of inflammatory markers, and absence of clinical or radiographic signs of recurrent infection at follow‐up. The use of inflammatory markers was interpreted as one component of the overall assessment rather than a standalone determinant.

**Figure 7:**
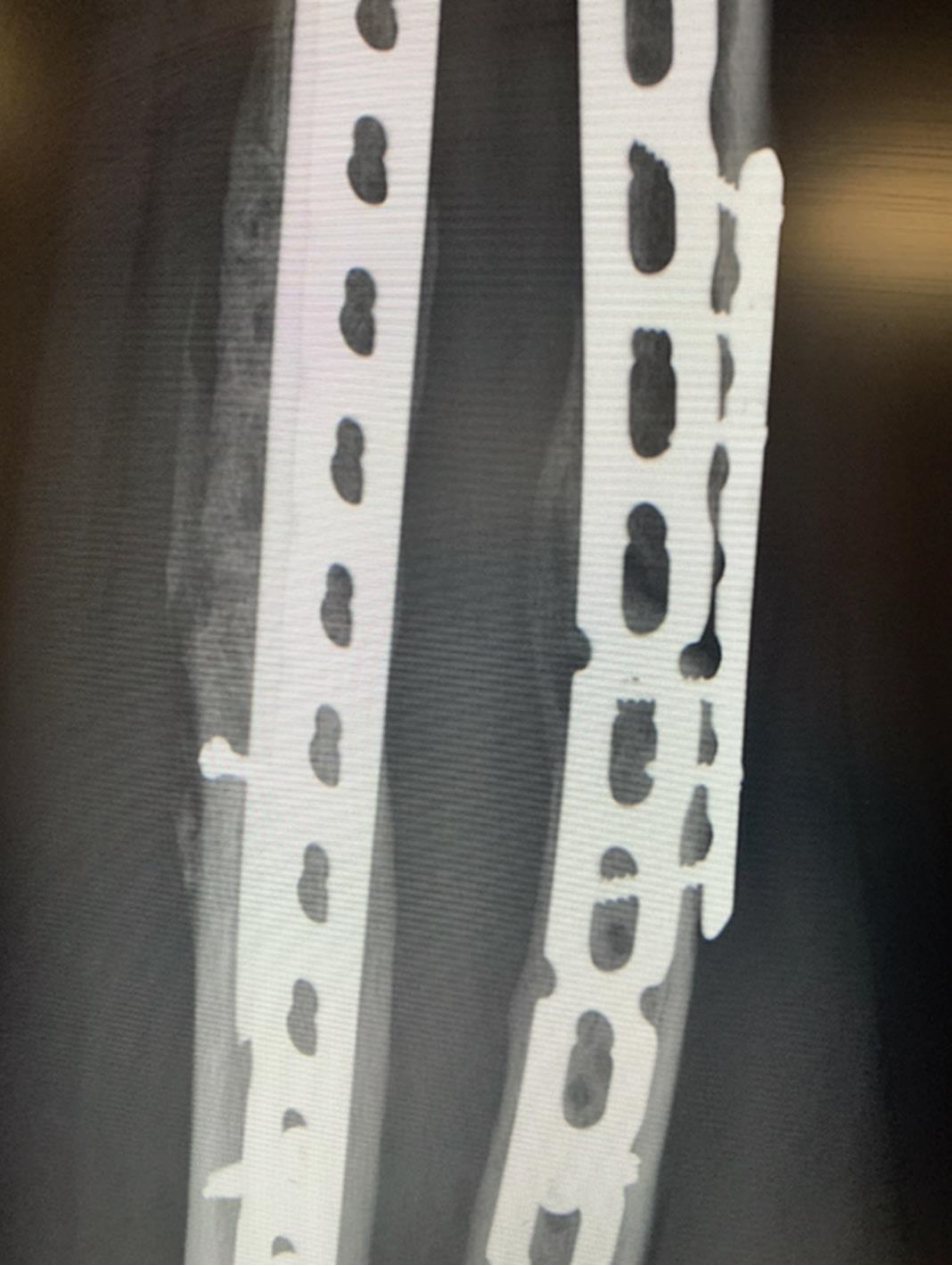
AP radiograph at approximately 6 months Stage 2 IMT, demonstrating early bridging and consolidation at the radius fracture and ulnar defect site, maintained dual plate fixation without hardware loosening.

At Stage 2 + 17 months (22 months postinjury), radiographs demonstrated mature bridging callus at both the radius and ulna on AP and lateral views, confirming solid union of both bones (Figure 8). Radiographic union was defined as bridging callus across at least two cortices on both the AP and lateral views in conjunction with pain‐free functional use of the extremity. All radiographs were assessed by the treating surgeon, who applied this prespecified definition. At this timepoint, prior to further elbow procedures, the patient demonstrated an elbow arc of motion of 60°–140° (flexion contracture of 60°, maximum flexion of 140°), forearm pronation of 70°, and supination of 75°, yielding a Mayo Elbow Performance Score of 65 out of 100 (fair), comprising pain (15/25), motion arc (20/25), stability (10/10), and function (20/25).

**Figure 8:**
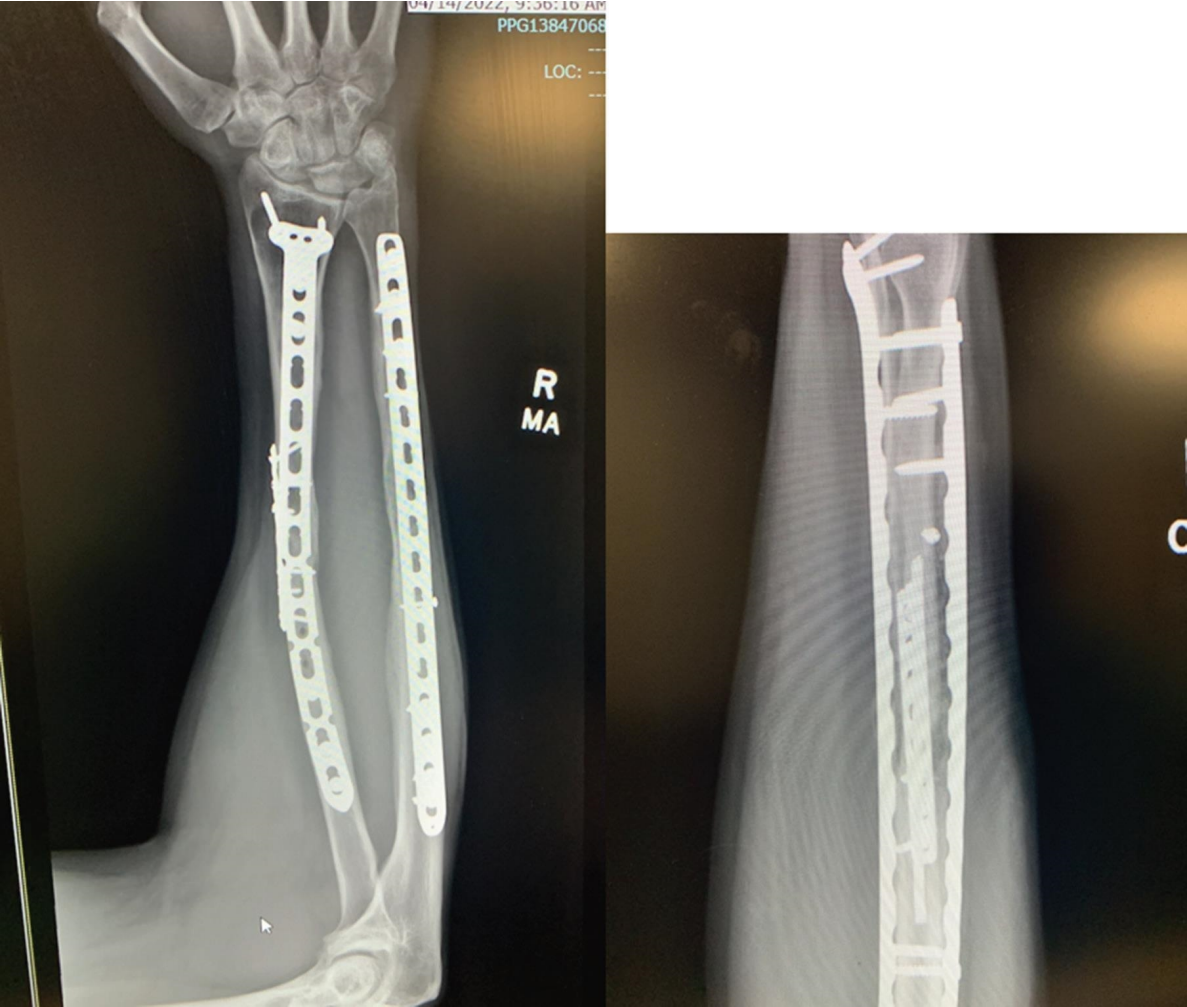
AP and lateral radiographs at approximately 22 months postinjury, demonstrating mature bridging callus at both the radius and ulna, confirming solid union of both bones and positive ulnar variance compared with preoperative and intraoperative imaging.

The patient subsequently underwent elbow contracture release via extensile capsulectomy at approximately 22 months postinjury, during which decompression of the radial and ulnar nerves was performed as a component of the surgical approach. A subsequent elbow procedure involving radial head resection was performed for additional osteoarthritis and fibrosis at an outside facility closer to the patient′s home; postoperative functional assessment following that procedure is not available for reporting.

## 3. Discussion

This case demonstrates successful induced membrane reconstruction of a 6‐cm critical‐sized ulnar bone defect in the setting of *S*. *marcescens* fracture‐related infection, with concurrent revision ORIF and bone grafting of a concomitant radial nonunion performed at Stage 2. It is important to note that the IMT was applied to the ulna only; the radius was managed with revision fixation and autogenous grafting at the same operative sitting. This distinction is clinically meaningful and informs the biomechanical discussion below.

The terms “Masquelet technique” and “induced membrane technique” are often used interchangeably throughout the orthopedic literature and refer to the same fundamental two‐stage reconstructive principle. Strictly speaking, the Masquelet technique as originally described incorporated soft tissue flap coverage as an integral component of the reconstructive sequence. The IMT is the more mechanistically precise designation, referring specifically to the biology of spacer‐induced membrane formation and graft incorporation. In this case, rotational fasciocutaneous flap coverage was performed at the index operation independently of the bone reconstruction; the staged spacer and grafting procedure for the ulna therefore aligns most precisely with the IMT designation. This distinction is noted given the widespread interchangeable use of both terms to encourage precision as standardized reporting becomes increasingly important for future comparative studies.

Mature bridging callus was noted at 6 months following the Stage 2 IMT procedure and confirmed at both bones at 22 months postinjury (17 months post‐Stage 2). This timeline reflects the cumulative complexity of the case: A two‐stage ulnar reconstruction performed in the setting of active osteomyelitis, with concurrent radial revision at Stage 2. Table 1 summarizes published reports of the IMT of the forearm [6–11]. Studies were identified through a structured search of PubMed and included if they reported outcomes of a two‐stage IMT to the radius and/or ulna. No restrictions were placed on study design or sample size given limited volume of forearm‐specific literature. This comparison is intended to contextualize the present case with the available literature and does not represent a systematic review. For forearm IMT applications, Walker et al. reported union at a median of 4.67 months post‐Stage 2 [6] and Bourgeois et al. at a mean of 4 months [7], though neither series involved the degree of infectious complexity or concurrent both‐bone management present here (Table 1).

**Table 1:** Comparison of published outcomes following the induced membrane technique for forearm bone defects. Studies include case reports and case series with varied etiologies including trauma, nonunion, osteomyelitis, and tumor resection.

Study	*N*	Defect (cm)	Location	Union (months)	Complications/outcomes
[8]	1	Not specified	Left ulna (atrophic nonunion); left radius (malunion)	6	None; full ROM achieved
[7]	6	Mean: 6.4 (4.8–11.0)	Forearm: distal radius (varied etiologies)	Mean: 4 (2.3–6.3)	1 nonunion; 1 unstable DRUJ; 1 S. aureus reinfection
[9]	7	Not specified	Forearm (radius and ulna)	Not specified	2 reinfections (29%)
[6]	9	Median: 4.7 (1.7–5.4)	Forearm (radius and/or ulna)	3–12	1 plate fracture requiring revision
[10]	10	Mean: 4.3	Forearm (radius and/or ulna)	Mean: 9.2	1 nonconsolidation
[11]	1	4.5	Forearm (radius and ulna)	2 (ulna)	Atrophic radial nonunion requiring repeat grafting
Present case	1	6.0 (ulna only); radius: nonunion (no defect)	Right ulna (IMT); right radius (revision ORIF + bone graft at Stage 2)	Mature union both bones at ~22 months postinjury	Positive ulnar variance post‐Stage 2; elbow contracture release at ~22 months; MEPS: 65 (fair) prior to elbow procedures; Serratia marcescens; culture‐negative at Stage 2

*S*. *marcescens* is an opportunistic gram‐negative rod with well‐characterized intrinsic resistance to ampicillin‐based agents and inducible AmpC beta‐lactamase activity, consistent with its isolation in the context of high‐energy freshwater and soil contamination. Both colony types were susceptible to TMP‐SMX, tobramycin, ciprofloxacin, gentamicin, and levofloxacin, confirming the appropriateness of the antibiotic strategy employed. Empiric doxycycline was initiated for a wound of unknown depth, then discontinued to preserve culture yield at the time of planned debridement. Following Stage 1, TMP‐SMX was initiated as culture‐directed oral therapy guided by intraoperative sensitivities, consistent with emerging evidence supporting oral step‐down strategies in bone and joint infections. Culture negativity and normalized inflammatory markers at Stage 2, as well as the absence of clinical or radiographic signs of recurrent infection through final follow‐up at approximately 17 months following cessation of antibiotics confirmed successful eradication prior to definitive reconstruction.

An important forearm‐specific biomechanical consideration arose in this case and warrants explicit discussion. In this patient, positive ulnar variance was observed following Stage 2 and appeared temporally related to the reconstructive strategy: The IMT spacer construct held the ulna at a fixed length throughout the staged procedure, whereas compression of the radius resulted in shortening at Stage 2 to achieve compression across the nonunion site during revision ORIF. This interplay between the two techniques produced a change in relative radioulnar length and consequently altered ulnar variance.

This dynamic is fundamentally different from what is encountered when applying the IMT to the tibia or femur. In the lower extremity, the tibia and fibula are linked by the proximal tibiofibular joint and the distal syndesmosis, both of which are relatively static articulations with minimal physiologic motion between the two bones. Length changes in either bone during reconstruction can be accommodated with static holding screws across these joints without producing critical changes in limb mechanics or joint function, as the degree of motion between tibia and fibula is inherently small.

The forearm, by contrast, functions as a coupled joint system. The radius and ulna are biomechanically interdependent through the proximal and distal radioulnar joints and the interosseous membrane, and forearm pronosupination depends on precise maintenance of relative bone lengths, bow, and rotational alignment. Fixing the ulna at a set length via the IMT construct while shortening the radius for nonunion compression may alter ulnar variance and, on the basis of established forearm biomechanical principles, could theoretically affect load distribution across the DRUJ. Whether the variance change observed here is clinically meaningful cannot be determined from a single case. In this case, the patient demonstrated no functional deficit in pronosupination at early follow‐up, suggesting that the degree of variance change was functionally tolerated; however, this is not universally guaranteed and warrants prospective monitoring. Surgeons planning simultaneous management of both forearm bones—whether through IMT, revision ORIF, or a combination—should anticipate the potential for radioulnar length discrepancy and consider its implications for DRUJ and elbow mechanics when sequencing the reconstruction.

The elbow flexion contracture that developed in this case arose as a consequence of severe periarticular soft tissue injury inherent to the index high‐energy trauma, rather than a failure of the bone reconstruction. Contracture release via extensile capsulectomy was performed, with decompression of the radial and ulnar nerves carried out as a standard component of the surgical exposure. The forearm reconstruction remained solidly united at the time of elbow surgery, clearly separating the elbow pathology from the bone reconstruction outcome. Functional status at the time of elbow contracture release was assessed using the Mayo Elbow Performance Score, which is a clinician‐assessed outcome instrument, which revealed a score of 65/100 [12].

Limitations of this report are inherent to the single‐case design, as these findings cannot be generalized to broader populations, and the absence of a comparator group limits any causal inference regarding the efficacy of the Masquelet technique relative to alternative strategies. External validity is further limited by the specific characteristics inherent to this report, including injury pattern, comorbidities, and treatment course. This may not be representative of all patients presenting with forearm nonunion. Functional outcomes in this report reflect clinical observation at a single timepoint rather than structured reporting on longitudinal assessment. In addition, a documented patient‐perspective statement could not be obtained, and validated patient‐reported outcome measures such as the Disabilities of the Arm, Shoulder, and Hand (DASH) or QuickDASH questionnaire may have strengthened the clinical relevance of the reported outcome. Furthermore, postoperative assessment following the patient′s subsequent elbow surgery at an outside facility is unavailable. As the extensile capsulectomy and later radial head resection would be expected to alter elbow function and forearm function, the ultimate functional outcome remains uncertain. In addition, radiographic union was assessed solely by the treating surgeon rather than by multiple independent reviewers, introducing potential subjectivity into the outcome assessment. Further, ulnar variance was assessed qualitatively. The biomechanical observation regarding radioulnar length is therefore descriptive and hypothesis‐generating. Larger prospective series with standardized outcomes reporting, microbiological data, and specific attention to radioulnar length management in combined forearm reconstructions are needed.

## 4. Conclusion

The IMT successfully reconstructed a 6‐cm critical‐sized ulnar bone defect in the setting of *S*. *marcescens* fracture‐related infection, with concurrent revision ORIF and bone grafting of a concomitant radial nonunion performed at Stage 2. Mature bridging callus was noted at 6 months and confirmed at both bones at 22 months postinjury with infection eradication confirmed at Stage 2. This case highlights a forearm‐specific biomechanical consideration distinct from lower extremity IMT applications: Because the forearm functions as a coupled joint system, fixing the ulna at a set length via the IMT construct while shortening the radius for nonunion compression may produce changes in radioulnar variance. Although this single case cannot establish a generalized biomechanical effect, we suggest that surgeons managing combined forearm bone pathology remain aware of this potential interplay when sequencing the reconstruction.

## Author Contributions

J.N.M. and A.B.C. contributed to manuscript revisions, writing, and editing. M.L., B.P., H.A., K.U.F., and C.J.B. contributed to manuscript drafting and editing. M.L. and H.A. additionally contributed to figure and table preparation. A.A.A. conceptualized and supervised the project, contributed to manuscript drafting, revisions, and editing, and serves as the corresponding author.

## Funding

No funding was received for this manuscript.

## Disclosure

All authors reviewed and approved the final manuscript.

## Ethics Statement

The manuscript follows the CARE guidelines. This case report was deemed exempt from formal Institutional Review Board (IRB) review per Piedmont Healthcare Policy #6408 regarding single retrospective case reports reporting the observation of single subjects. Written informed consent was obtained from the patient for publication of this case report. All patient data were deidentified in compliance with HIPAA.

## Consent

Written informed consent was obtained from the patient for publication of this case report and accompanying images. A copy of the written consent is available for review by the editor in chief upon request.

## Conflicts of Interest

The authors declare no conflicts of interest.

## Data Availability

The data that support the findings of this study are available on request from the corresponding author. The data are not publicly available due to privacy or ethical restrictions.
